# Book Reviews

**Published:** 1820-06

**Authors:** 


### CRITICAL ANALYSIS

OF

### RECENT PUBLICATIONS, IN THE DIFFERENT BRANCHES OF MEDICINE AND SURGERY.

"I would have men know, that, though I reprehend the easie passing over of the causes of things, "by ascribing them to secret and hidden vertues and properties; (for this hath arrested and laid "asleepe all true enquiry and indications;) yet I doe not understand but that, in the practical "part of knowledge, much will be left to experience and probation, whereunto indication cannot "so fully reach: and this not only in *specie*, but in *individuo*. Yet it was well said, *Vere scire* "*esse per causas scire*."—BACON.

*A Treatise on Nervous Diseases.* By JOHN COOKE, M.D. F.A.S. Fellow of the Royal College of Physicians, and late Physician to the London Hospital. In two Volumes. Vol. I. *On Apoplexy, including Apoplexia Hydrocephalica, or Water in the Head; with an Introductory Account of the Opinions of Ancient and Modern Physiologists, respecting the Nature and Uses of the Nervous System.* Read at the College, as the Croonian Lectures of the Year 1819. 8vo. pp. 469. Longman and Co. 1820.

THE author remarks, in the Preface to this work, that "it was the opinion of a late eminent physician, that more real service may be rendered to medicine by the illustration of what is already known on the subject, than by any attempts to promulgate new theories or new modes of practice. Impressed with the justice of this opinion," he continues, "and the propriety of acting upon it, I have taken considerable pains in endeavouring to collect, to arrange, and to communicate, in plain clear language, a variety of useful observations from the best authors, both ancient and modern, respecting the principal diseases of the nervous system. If the example which I have presumed to set should be followed; if persons, better qualified for the task than myself, would investigate other important diseases on a similar plan, a system of medicine would be formed which might prove eminently useful, both by lessening the labours of the student, and affording practical facilities to persons actually engaged in the duties of the profession." The present volume is the first result of the author's exertions consonant to the views above designated, and it is his intention to treat, in a second one, of palsy and epilepsy. The treatise before us is a good exemplification of the correctness of the sentiments expressed in the latter part of the above cited paragraph; but when we contemplate, in the *Anatomie Génerale* of BICHAT, what has been produced in physiology, in our own days, by the genius of one man devoted to original researches, and when we consider that pathological anatomy has not yet emerged from its infancy, we are not inclined to rate as low as the author appears to do, the value of original investigation, or of attempts to reason better than men have hitherto done on the nature of such phenomena as have already become subjects of observation. It is, however, the character of the work before us, not the propriety of those views, that should here be the object of our consideration: to that we therefore turn.

The introductory account of the opinions of ancient and modern physiologists respecting the nature and uses of the nervous system, is, of course, not a proper subject for analysis in this place. The only remark we shall make on it is, that, though it be imperfect, it contains more interesting matter than we expected to find in so small an extent of space: a satisfactory exposition of so extensive a subject, would itself necessarily occupy several volumes; and it is the pathology of the nervous system, rather than its physiology, of which the author expressly purposes to treat. The treatise on Apoplexy is, indeed, not calculated for regular analysis, and we should, perhaps, best perform our critical functions, in stating in a few words our judgment respecting the manner in which the author has fulfilled his intentions; but, as there are but few points on which he has not adduced his own observations and opinions, we shall take a rapid view of the whole, in order that the original parts of it may be brought forward in a clear and orderly manner.

The term *apoplexia* was employed by the Greeks, and is still used, the author remarks, to denote a disease in which the patient falls to the ground, often suddenly, and lies without sense or voluntary motion: GALEN, however, whose physiological views were astonishing, for their correctness, on many occasions, excepts that of *respiration.* PAULUS ÆGINETA, in his description of it, took care to state that it is without fever; as if he saw the necessity of making a distinction between effects apparently similar, but dependant on very different causes. The Greek writers, in general, seem to have considered apoplexy and palsy as diseases of the same nature, as Dr. Cooke shows by his citations. ARETÆUS says, apoplexia, paraplegia, paresis, and paralysis, are all of the same kind, and consist in a defect of sensation, or of motion, or of both. Apoplexy is a palsy of the whole system; of mind, of sense, and of motion. Galen says, when all the nerves have lost sense and motion together, the disease is called apoplexy: when this happens to a part only, whether the right or the left, it is called palsy. But we find some authors speak of apoplexy of the tongue, the arm, and the leg. CELSUS also uses the terms apoplexy and paralysis synonymously. It would appear from some passages in the writings of HIPPOCRATES, that he used the word ἀφωνια, to designate apoplexy without complication with palsy.

In the writings of modern nosologists there is a great variety of definitions. Sauvages denominates it a most profound sleep, with stertorous respiration; and Linnæus describes it in almost the same terms. Cullen says, "apoplexy is a disease in which the whole of the external and internal senses, and the whole of the voluntary motions, are in some degree abolished, whilst respiration and the action of the heart continue to be performed. By its being an affection of the whole of the power of sense and voluntary motion, he says, we distinguish it from palsy; and, by its being with the continuance of respiration and the action of the heart, it is distinguished from syncope." He excludes stertorous breathing from the necessary phenomena, as well as Young and Portal. Dr. Cooke thinks it may be thus defined: "It is a disease in which the animal functions are suspended, while the vital and animal functions continue; respiration being generally laborious, and frequently attended with stertor."

This definition does not, we think, clearly mark its difference from common profound sleep with sufficient clearness and precision; and it is, perhaps, not possible to found one on the phenomena presented by the body externally, precisely applicable to it, that will mark this difference; as Boerhaave had indeed remarked. But we are beginning to relinquish nosology for nosography; that is, a useless subject, or one only fit for scholastic exercises, for one that is really a guide to the clinical practitioner.

In a fit of apoplexy, says the author, the patient falls to the ground, and lies as if in a deep sleep, from which he cannot be roused. In the strong paroxysm, persons are said to lie entirely deprived of sensation and motion; but the power of moving is occasionally apparent, and we cannot be certain that the power of feeling in these cases is wholly abolished. Dr. Cooke continues to remark, that he has seen patients in this disease shrink on being cupped, and move their hands towards the head, as if feeling uneasiness there. In the perfect, or strong apoplexy, the respiration is generally much impeded; but, although laborious, it is often, in the beginning of the paroxysm, slow and regular: in the middle, and towards the end, when the disease terminates fatally, it becomes frequent, weak, and irregular. Galen, Boerhaave, and Portal, measure the intensity of the disease by the degree of laborious or stertorous respiration; and Dr. Cooke also says, that, in all the cases of strong apoplexy which he has seen, the respiration was laborious, slow, and stertorous, in the beginning of the paroxysm; and in those which proved fatal, this symptom, as far as he can recollect, remained, even when the breathing had become weak and irregular. In the strong apoplexy, a frothy saliva, or foam, is frequently excreted from the mouth, which is sometimes blown away from the lips with considerable force; and this phenomenon has been considered by some authors to indicate a violent disease.

The pulse, especially in the beginning of the paroxysm, is generally described as being regular, slow, full, sometimes hard; but, in a few hours, if no amendment appear, it is said to become irregular, frequent, and weak. It has struck us that the frequency of the pulse has commonly increased in a direct ratio to the slowness of the respiration. Thus, the pulse is often about seventy when the number of inspirations is fifteen in a minute; and, as this number has fallen to eight or ten, the pulse has proportionately rose to 80 or 90. The author adduces a very interesting observation from Dr. CHEYNE, respecting the continuance of the irritability of the heart after the cessation of respiration. "Sitting," he says, "with my finger over the artery of a person who died of apoplexy, I distinctly felt the pulse beat after the last expiration." Both the latter phenomena are explicable, by the consideration that the heart does not depend on the influence of the brain for its nervous energy, whilst the actions necessary for respiration derive their motive influence from that organ. The other functions of nutrition are carried on with moderate activity: the contents of the intestines and urinary bladder are, it is true, commonly retained; but this is because the voluntary muscles of the belly cannot be called into action, the aid of which is necessary for their evacuation; and, for the same reason, vomiting is not excited without great difficulty, and it cannot be at all produced in many cases; though it is evident that the stomach retains its ordinary sensibility, for the medicines commonly employed to produce it leave marks of irritation, and often of severe inflammation, in that organ after death.

Dr. Cooke says, that "the face and whole body are sometimes cold and bedewed with clammy sweat; but more frequently the temperature of the skin is higher than natural, and is accompanied with copious perspiration." These phenomena are inexplicable on any other principles than the distinctness of the nervous system regulating the functions of nutritive or organic life, from that directing those of relative or animal life. Sense and voluntary motion are here nearly or totally suspended, and the actions of the organic system proceeded with a degree of activity even above that of the ordinary standard; and the heat of the body is increased, though respiration be lessened in frequency.

Fever is mentioned in the list of symptoms of this disease by some writers; and Sir GILBERT BLANE says, that blood drawn from persons in the apoplectic paroxysm is almost always covered with the inflammatory crust: but Dr. Cooke believes that fever seldom accompanies it. Portal, and those who mention it, appear to confound the stupor which accompanies inflammation of the brain with simple apoplexy.

The eyes are most commonly quite closed; the cornea is dull and glassy, and the pupils are dilated in the generality of cases. But Dr. Cooke has seen them greatly and permanently contracted, in some instances almost to a point, and a physician of eminence of his acquaintance has observed the same phenomena. Aretæus and Cheyne appear to be the only authors who had previously made the same remark. The teeth, the author next states, are often closely locked together. These two last circumstances will not surprise us, when we consider how intimately and directly the iris and the muscles of the lower jaw sympathize with the alimentary canal, by means of the lenticular and upper cervical ganglions; and we know that irritation of the stomach and intestines often accompanies apoplexy, or is produced in the course of the disease by the medicines given to produce vomiting or evacuation of the bowels, without considering that neither of these actions can be effected without the aid of the abdominal muscles, which cannot be obtained whilst the state of apoplexy is very profound. It is a curious thing, too, that the mucous membrane of the stomach and intestines appears here to be even more irritable than in the state of health: it would seem that the vitality of those organs is increased in consequence of the want of expenditure of it by the organs subservient to volition. The author says, he believes the secretions are seldom much altered in this disease.

The paroxysm generally lasts from eight to twelve, twenty-four, or forty-eight, hours; sometimes for a still longer period. Genuine apoplexy, Dr. Cooke thinks, seldom destroys life in less than one or two hours. Sudden deaths, he remarks, are very commonly ascribed to apoplectic seizure; but they probably depend on some other affection. The effects of inhalation of carbonic acid and other deleterious gases, should not, he properly considers, be confounded with apoplexy. When it does not prove fatal, it usually terminates in paralysis. The strong apoplexy almost always ends in death.

With respect to the appearances on dissection after death from apoplexy, Dr. Cooke has advanced nothing original; and we were not a little surprised to find no mention made of the important observations of Rouchoux and Riobe, of which Wepfer, Brunner, and Morgagni had a glimpse, on the formation of cysts round the coagula of blood in the substance of the brain.* They serve to explain many important circum-

* See the Number of this Journal for January 1819, for an account of those observations in the Historical Sketch of the Progress of Medicine.

stances in the consequences of apoplexy, and indicate some useful practical measures, as we hinted in our review of M. Lerminier's paper, in a recent Number of this Journal.

A multitude of authorities are adduced by the author, to show the influence of a wet and cold constitution of the atmosphere in the production of apoplexy; a circumstance we had occasion to point out in our Reports of Diseases during the winter of 1818-1819. On discussing the point of the influence of gluttony and spirituous potations in the production of apoplexy, the author seems not disposed to attribute so much to the latter agent as Dr. Cheyne and some other authors, unless it be carried to excess; and, except this be the case, he thinks that apoplexy seldom occurs amongst the labouring poor. Our observation has led us to form a different opinion; and, when Dr. Cooke states that he does not recollect "a single instance of the occurrence of apoplexy at the London Hospital during an attendance there of above twenty years," we are disposed to consider that this has arisen, not from the poor being but rarely affected with the disease, but because patients in a fit of apoplexy are not usually removed to hospitals. Several practitioners whose duties call them to attend parish paupers, have informed us that the disease is common amongst them; and we have often remarked, in the dissecting-rooms of the medical schools in different parts of Europe, the great proportion of cases in which, especially during the latter part of autumn, old subjects have apparently died from apoplexy: effusion of blood in the cranium being present, generally with ossification of the cerebral arteries. Men who have spent their life in hard labour are more subject to ossification of the arteries on the approach of old age, than those of the upper ranks of society; and this cause appears to nearly equal, in regard to the production of apoplexy, the great luxury in diet of persons in general of the latter class.

When treating of the exciting causes of the disease, Dr. Cooke states that, as far as he has been able to compare symptoms with appearances on dissection, the sudden apoplexy seems always more or less connected with an effusion of blood. Effusions of serum are also amongst the exciting causes; but tumors, which are slow of formation and growth, can only be admitted into the list of remote causes. They may give occasion to head-ach, vertigo, lethargy, stupidity, blindness, epilepsy, &c. and they may, by their increase, in time, produce apoplexy, but not the sudden disease. Although an effusion of serum may give rise to apoplexy by pressure, Dr. Cooke says he is persuaded that an effusion of blood is, in a very great proportion of cases, "the exciting causes of the sudden strong disease." Our experience has led us to agree with the author, as regards the "sudden strong disease," especially in old persons; but apoplexy that has ensued after a few days of vertigo, head-ach, &c. has appeared to us to present more frequently serous effusion after death than extravasated blood; and it is in cases of this kind that no effusion whatever is discovered on dissection, in some instances, as Drs. Abercrombie and Cheyne lately, and the physicians of Breslau, HOFFMANN, CASIMIR MEDICUS, and RAHN, had long since observed, when the disease soon terminates fatally. These seem to be the cases which led some of the older writers to constitute a species of apoplexy which they termed *nervous*.

The author passes in review all the most important remote causes of this disease with great care and critical acumen; and he comprises in this chapter the theories of death from hanging and drowning; which we shall transcribe, as of considerable practical importance, and because many medical practitioners seem to have no determinate opinions on this subject: they will, also, give the reader an idea of the author's manner of discussing the points he takes into consideration.

"Some physiologists are of opinion, that hanging produces death by inducing apoplexy. M. Portal says, persons who are strangled do not die of suffocation or want of respiration; they perish from apoplexy. In proof of this, he observes, that Morgagni, Lieutaud, and other great anatomists, have mentioned in such cases a large quantity of blood found in the vessels of the brain, or in the cavities of that viscus, either without water or mixed with water. He adds, water is sometimes found limpid and unmixed with blood. The examination of the bodies of persons who had been hanged, says M. Portal, which were formerly brought to us at the Jardin des Plantes for our lectures, furnished the same results.* Dr. Dejean, professor of medicine at Caen, convinced himself, by experiments made on living animals, that the result of strangulation was apoplexy, and that it was the effect of a congestion of blood in the brain.† Mr. Brodie informs me, that he found a large quantity of blood extravasated in the brain of a man who had been hanged; and that Dr. Hooper has in his possession a preparation of the brain of a person who died in a similar way, which exhibits a great deal of blood effused among the membranes. Some modern physiologists, however, do not agree in sentiment with M. Portal. Dr. Curry mentions an experiment, hereafter to be related, which appears conclusive on the subject.

"It was formerly a common opinion, that drowning kills by inducing apoplexy; but Cullen, Hunter, Goodwyn, and others, maintain, that death from drowning entirely depends upon the obstruction of respiration, which takes place under these circumstances. Dr. Cullen, in his letter to Lord Cathcart, concerning the recovery of drowned persons, 'considers it probable that the death which ensues,

* Portal, p. 300. † *Ibid*, p. 302.

or seems to ensue, in drowned persons, is owing to the stoppage of respiration, and to the ceasing, in consequence, of the circulation of the blood, whereby the body loses its heat, and with that the activity of the vital principle.' Mr. Hunter says, 'the loss of motion in drowning arises from the loss of respiration;' and he thought that this was the first cause of the cessation of the motion of the heart. Dr. Goodwyn agrees in opinion with Dr. Cullen and Mr. Hunter. Dr. Goodwyn made many experiments with a view to ascertain the effects of submersion upon living animals, from which he draws the following conclusions: 'That a small quantity of water commonly passes into the lungs in drowning; that the water enters into the lungs during the efforts to inspire, and, mixing with the pulmonary mucus, occasions the frothy appearance mentioned by authors; that the whole of this fluid in the lungs is not sufficient to produce the changes which take place in drowning: whence it follows,' he says, 'that the water produces all the changes which take place in drowning, indirectly, by excluding the atmospheric air from the lungs, not directly, by entering into the cavity of the lungs.'

"Dr. Goodwyn takes great pains to investigate the cause and manner of death from drowning, and is by no means disposed to consider the disease to be apoplexy which is produced by submersion. Under these circumstances, death, he thinks, takes place, because the blood cannot receive oxygen from the air by respiration, without which the heart cannot be stimulated to action. 'When the pulmonary blood,' says Dr. Goodwyn, 'is no longer fitted to excite the sinus venosus and auricle to contraction, they receive it into their cavity, and remain at rest. As soon as they cease to contract and propel the blood to the head, all the intellectual operations cease, and voluntary motions are suspended, and the external signs of life disappear.' The disease, Dr. Goodwyn thinks, is in the blood, and consists in the presence of this black blood in the left side of the heart and arterial system, and might with more propriety be named melanæma.'* Under melanæma, as a genus, Dr. Goodwyn would place the diseases brought on by hanging and drowning; as, *melanæma à suspensione, melanæma à submersione.*

"Dr. Curry thinks that, in hanging, as well as in drowning, the exclusion of air from the lungs is the immediate cause of death. 'From the great accumulation of blood in the vessels of the head,' says Dr. Curry, 'many have been of opinion, that hanging kills chiefly by inducing apoplexy; but the following ingenious experiment, made at Edinburgh many years ago by Dr. Monro, sen. clearly proves that the exclusion of air from the lungs is the immediate cause of death. A dog was suspended by the neck with a cord, an opening having been previously made in the windpipe below the place where the cord was applied, so as to admit air into the lungs. In this state he was allowed to hang for three-quarters of an hour, during which time both the circulation and breathing went on. He was then taken down, without appearing to have suffered much from the experiment. The cord was

---

now shifted from above to below the opening made into the windpipe, so as to prevent the ingress of air into the lungs; and the animal being again suspended, he was completely dead in a few minutes.'*

We cannot attempt to abridge even the most important parts of this chapter, it being itself a concise view of matters comprising a multitude of important considerations; and it peremptorily calls forth the expression of our sentiments (which we generally endeavour to render apparent by what we produce from an author, rather than in express terms), of the great merit and practical utility of the work. The research on which the author's history of the disease has been founded, is original and very extensive; but we cannot say quite complete; as no notice is taken of some important German authors who have written in their vernacular language. This fault runs through the whole work; and, as of late years most of them have ceased to use the Latin, a great deal of important matter, in clinical observations, has escaped the author's attention. We must however expressly state, in respect to the last chapter, that Dr. Cooke has not noticed the connexion of apoplexy with hypertrophy of the heart, mentioned in the Proëmium to the present volume of this Journal; a connexion of disease that, it appears to us, possesses a remarkable degree of interest, and of which the knowledge is of considerable practical importance.

The *distinctions* of apoplexy are next considered. The author says, "Dr. Cullen's division of apoplexy into species, so far as they depend upon evident causes leading to a corresponding mode of treatment, such as the *traumatica* and *venenata*, may, perhaps, be safely and properly admitted; but, when distinctions are made upon a presumption only of the knowledge of causes, and yet lead to a specific practice, as in the *sanguinea* and the *serosa*, they should not, I think, be received without a very careful examination of the grounds on which they are made." The doubt of the propriety of this division is, we think, rendered more evident by the fact, that blood and serum are both effused in many cases: we often find the ventricles filled with a sero-sanguineous fluid; and probably the same irritation of the arachnoid that in one degree gives rise to exhalation of only serum, will, on being increased, give occasion to sanguineous effusion. The best distinction would perhaps be, into that from rupture of blood-vessels, and that from exhalation of fluids from their capillary extremities, developed in the arachnoid membrane, consequent on irritation of them: the former giving rise to the sudden disease, and ordinarily depending on ossification of the arteries; the latter coming on more slowly, and depending on the common causes of irritation,

* Curry's Observations, p. 71.

and attacking persons less far advanced in life than the former. We may add, that effusions of pure blood in the ventricles originally never takes place: when it is present in them, it will be found to have made its way there from the substance of the brain, where it was poured out.

With regard to the *apoplexia hydrocephalica*, or *hydrocephalus internus* of Cullen, Dr. Cooke says, "I wish to observe, that it appears to be a complaint very different from the true apoplexy, both as to its nature and treatment, and ought to be considered as a distinct and separate disease." Yet, we think, it would be very difficult to say in what the precise difference between this and *serous* apoplexy consists. They both seem to depend on, either irritation of the exhalents of the arachnoid membrane, or venous congestion, preventing the due absorption of the serum ordinarily exhaled; and hence, from these causes respectively, acute and chronic hydrocephalus.

After passing in review the classifications of the nosologists and the opinions of the greater part of the most eminent writers, Dr. Cooke concludes with saying,

"On the whole, if we admit the distinction of apoplexy into the sanguineous and serous, I think, we must also admit that the serous apoplexy very seldom occurs; and I am of opinion that, even in very old persons, of leucophlegmatic habits, pale countenance, small pulse, and other marks of serous apoplexy, if the disease come on suddenly, it ought to be considered as probably arising from an effusion of blood within the cranium."

We may add to this, that when the apoplectic attack has been preceded for some days by head-ach, vertigo, &c. that it will commonly be found to have arisen from serous or sero-sanguineous exhalation into the ventricles of the brain, or on the surface of its hemispheres. The *sudden* disease most commonly attacks old people; and, as we before remarked, it appears to result, in a great proportion of cases, from rupture of a blood-vessel, commonly dependant on ossification of its parietes.

The *diagnosis* and *prognosis* are next considered. On the former point, such an abstract as we could here adduce of the author's discussion, would not convey more useful information than what is indicated in the foregoing observations. On the latter point, the author protests against the decision of some writers, that the strong disease itself is always fatal: he has himself seen more than one instance of recovery from it; and he inserts a communication from Mr. Astley Cooper on this subject, who says:

"The dissections which I have made of cases of apoplexy, and extravasations of blood upon the brain from accident, have led me to the belief that the effused blood never becomes absorbed, but that the brain gradually acquires the power of bearing its pressure; and that thus the symptoms which are produced at the first moments of general extravasation gradually diminish.

"I will give you instances of these extravasations. My friend and pupil, Mr. Saunders the oculist, had repeated slight apoplectic attacks for many months before his death, of which he apparently recovered; but at length he died from a sudden and large extravasation of blood into one of the ventricles of his brain. Upon examination of his head, besides the great extravasation above mentioned, several streaks of coagulated blood were found in the pons varolii and in the cerebellum, the colour of which was so different from the recent extravasations, as clearly to indicate that they had been long effused.

"The other example is, that of a gentleman who fell from his horse, struck his forehead violently, and was taken up comatose. He recovered from these symptoms, and appeared to be well, excepting that he had a slight defect in vision. Three months afterwards, from improvident conduct, he brought on symptoms of inflammation of the brain, of which he died; and, upon examination of his head, a large coagulum, which I have preserved, was found deeply embedded in the anterior lobe of the cerebrum, opposite the part at which he had received the blow, and which had the colour of blood long retained in an aneurismal sac."

The observations of Rouchoux and Riobe, to which we have already referred, and the more recent researches of Serres and Bricheteau, present more favourable results; for they show, indubitably, that coagula of blood are often absorbed from the brain, by the medium of the cyst formed around them, of which we have spoken. We cannot accompany Dr. Cooke through his remarks on the observations of other authors, but we shall adduce those which are the results of his own experience.

"When the pulse," he says, "after having been slow, strong, and full, becomes quick, weak, and intermitting, especially in conjunction with other unfavourable signs, we may conclude that the disease will soon terminate fatally.

"Among the dangerous signs in apoplexy, many authors mention a dilated state of the pupil of the eye; but the contracted pupil, which I consider to be a still more dangerous appearance, has been scarcely noticed. I am of opinion, that this ought to be reckoned among the very worst symptoms of the disease. I never knew a person recover from apoplexy when the pupil was greatly contracted. My opinion on this subject is confirmed by that of Sir Gilbert Blane and Dr. Temple.

"Cold and profuse sweats are very unfavourable symptoms in apoplexy. Hippocrates thinks, that sweat coming on after difficulty of breathing, is a fatal sign, and coldness and torpor dangerous.* Etmuller adopts this opinion, and considers this sweating as indicative of a failure of the vital powers, and not a natural perspiration.† When we

---

† Etmuller, p. 907.

have reason, from the sudden accession of the disease and other circumstances, to think that a considerable vessel in the brain is ruptured, we may almost despair of the patient's recovery."

Dr. Cooke makes Hippocrates state that, when fever comes on in patients of this disease, a solution of it takes place, (Ἐν ἀυτοῖσι (αποπληκτικοῖσιν) δὲ παλιν τοὺτοισιν ἤν πυρετὺς ἐπιγένηται, λύσις, *Coac. prenot.*;) but this judgment is so contrary to what modern experience seems to indicate, that we are disposed to think Hippocrates meant this to relate to paralysis, (in which it is more evidently true;) and we have already shown that he speaks of apoplexy of the tongue, the arm, the leg, &c.; and, what favours the notion we propose, is, the cause Hippocrates assigns for the cases alluded to in the passage cited by Dr. Cooke, (just transcribed,) being that to which the ancients generally attributed paralysis. In the sentence immediately preceding that above cited, they are designated as dependant ἐπι τῇ δυσφοριν: and, he adds, *Ta exaiphnes apoplectika lelymenos epipyretenanta chrono, olethria.*

With respect to the hopes that may be entertained of recovery at different periods of the disease, Dr. Cooke says,

"If the strong apoplexy has continued for even half that time [one day], I believe it almost always terminates in death. If the patient does not show symptoms of amendment soon after the employment of the most powerful means, a fatal termination of the disease may be expected.

"If the pulse sink and intermit, if coldness of the extremities, with cold clammy sweats, come on, and the power of respiration greatly diminish, we may predict that dissolution is inevitable, and fast approaching."

The chapter on the *treatment* of apoplexy is another of which we are unable to give an useful abstract. The author himself much favours the use of blood-letting, both as a preventive and remedy: blood should, he says, in the strong disease, "be evacuated speedily and freely, generally and topically;" an opinion that is supported by almost all those authors who have written from due reflection on actual observations: but Dr. Cooke is far from advising the use of it to such an extent as some others have done. He says, "I would not venture to persist in the abstraction of blood, if, after free and repeated bleedings, there was no apparent advantage; and, *a fortiori*, if symptoms of debility should supervene." A case related by Lancisi outvies, however, any thing that even the most zealous favourers of blood-letting have of late adduced. A man, he says, fifty years of age, who indulged in the pleasures of the table to excess, very fat, and of sedentary habits, experienced, for about a month, such a heaviness and somnolency, that he would fall asleep even when counting his money: he was evidently threatened with apoplexy, when he lost in one night eleven pounds of blood by nasal hemorrhage; he was not weakened by it, and felt somewhat lighter. Four days afterwards, he lost four pounds more blood, and was restored by it to good health. Some cases of this kind, not less interesting, were related by Dr. KINGLAKE, in the last volume of this Journal.

The author then passes in review the other remedies that have been employed or proposed for this disease. None of them has given occasion to so much disputation on their propriety as emetics: we shall transcribe the conclusions of Dr. Cooke on this point.

"If I were to give an opinion on this very important question, I would say, that I think our practice in this respect ought to be guided by the particular circumstances of each case. In the strong apoplexy, there may be danger of determining too much blood to the head by the act of vomiting: I therefore would not venture to prescribe an emetic till the safer remedies had been unsuccessfully employed. Although we do not precisely know how the brain is affected during the act of vomiting; and, although we are informed by Dr. Bryan Robertson and others, that emetics may be safely given in hemoptysis and other hemorrhages, I think that vomiting should not be excited, in the strong apoplexy, till depletion had been tried in vain: but if, after free and repeated evacuations of blood, both general and topical, and the administration of clysters and other revellents above mentioned, no signs of amendment should be perceptible, I would endeavour to excite the action of the vis medicatrix naturæ, by the exhibition of an emetic of speedy operation, such as the white or blue vitriols. In favour of the practice I am now venturing, under certain circumstances, to recommend, I would observe, that some instances might be given of restoration, from even the strong apoplexy, on the exhibition of an emetic; and I myself have witnessed one case of recovery from the disease in a somewhat milder form by this remedy, when bleeding, &c. had been prescribed without any good effect."

*Lethargy*, *coma*, *carus*, and *cataphora*, are then the subjects of discussion; but nothing new, in the way of distinction or nosological arrangement of those affections, is proposed by the author; and he remarks, that but little has been said about either their nature or treatment that is not to be found in the writings of the ancients.

On *apoplexia hydrocephalica*, we find hardly any thing original of importance; and the history of this affection is much less perfect than that of apoplexy. Many valuable works are not noticed, and none of those of the later German physicians, which we referred to in our review of Dr. Cheyne's Essays on this disease. The works of Goelis, Formey, Hopfengartner, Portenschlag-Ledermayer, and several others, would have furnished some very important observations respecting the most frequent remote causes of the disease, to place in opposition to those which seem to have been, as we think, rather too exclusively regarded by most of our modern writers.

---

*An Inquiry into certain Errors relative to Insanity; and their Consequences; Physical, Moral, and Civil.* By George Man Burrows, M.D. F.L.S. Fellow of the Phys.-Med. Society of the University of Erlangen; Member of the Royal Medical Society of Edinburgh; of the Atheneum of Medicine of Paris; of the Mineralogical Society of Jena, &c. 8vo. pp. 320. London, 1820. T. and G. Underwood.

There are some erroneous notions, even of the grossest kind, of which we can not only discern the origin and development, but which are such as any individual perceives he might himself have formed and has apparently avoided only by the intervention of some idea that has led him to discern the fallacy of the train of thought whence they arose: but the notion that such a derangement of the manifestation of the intellectual faculties as constitutes what is termed insanity can have a solely metaphysical origin, is one that baffles all our efforts to trace to any precise ideas or series of connected reasonings. This error, however, became very prevalent after the speculations of the scholastic metaphysicians were adopted in physiology, and it has exerted a more baneful influence on the practice of medicine than any other that has been assumed by physicians, and permitted to serve as a guide for their conduct. The ancients never imagined such a metaphysical disorder, but sought for the causes of insanity in deranged sensibility, depending on either original organic lesion of the organ of thought, or sympathetic influence from disease of some of the other viscera. They did not, therefore, as we have done, send the victims of it to solitary and close confinement, without attempting to cure a disease that is as proper a subject for the application of the materia medica as any of those for which drugs are ordinarily administered. But the evils of the notion above mentioned did not rest here; for, in attributing the malady to a supposed cause, the true and most common ones have been overlooked. We take our children at an age, when to eat the food presented to them, and to exercise their limbs, should be their only voluntary exertions, and we will have them not only acquire a multitude of ideas incongruous with their state, and utterly useless to them, but they must also be made to reason on abstract moral points that are calculated to lead even adult persons to madness, and then we wonder why so great a proportion of them die in their infancy from hydrocephalus, from the consequence of inordinate excitement of the brain. Those who survive are next submitted to the discipline of our schools; and here, especially since the invention of the art of printing, the process for effecting that morbid development of sensibility and destruction of muscular vigour, that fills society with fools that nature never makes, and mad-houses with lunatics, is progressively pursued, to be completed by the vivid and incessant moral emotions which every one must suffer who enters that train of social life which is vainly said to be proceeding towards the grand perfection of human nature. Thus, our organic powers languish; the brain and nervous system become the chief centre of vitality; sensibility is inordinately exalted; and then, by any unusual excitement, the ordinary moral relations of man with external nature become so far destroyed, as to constitute the state which is qualified with the term madness.

The ancients, whose confined, but often profound, views we are very ready to despise, because the extent of our ideas has been so considerably increased since the invention of the art of printing and the improvement of machinery, though they attributed a physical origin to insanity, were not neglectful of the influence of moral impressions in its alleviation. In Egypt, lunatics were led to temples, where all the objects capable of producing varied and agreeable sensations were assembled: such were the means employed to seduce their imagination, to avert dominating ideas by a multitude of impressions succeeding each other with rapidity; the whole of which were qualified constantly to occupy their sensibility, and thus to leave none for distressing ideas or painful remembrances. Asclepiades remarked, that, amongst all the means calculated to restore reason to men affected with phrenzy or melancholy, music was the most successful; and Dubuisson, in our own times, is disposed to think hardly less favourably of its powers. Some difference may be expected in the results of this practice, and that by which patients are delivered up to confinement, solitude, and perhaps restraint of almost every voluntary action; and thus the whole of their sensibility is exercised in the contemplation of the painful ideas which, in many cases, have given rise to their malady; or it is accumulated during intervals of torpor, to be developed in exciting furious emotions on the impulse of the slightest impressions.

These errors in the treatment of the insane have, at least until of very late years, been generally prevalent, and are indeed not yet by any means totally avoided; and it is the object of the work before us to expose the principal evils that have resulted from them.

In order to enable the reader to form an idea of the contents of this production, we shall, in the first instance, give a statement of the objects of the several sections of which it is composed, and then make some remarks on the manner in which the author has discussed them individually.

After some preliminary observations, the intent of which is to state the advantages that must result from the disposition to regard insanity as a malady of physical origin that is now become prevalent, and from the efforts of the legislature to ameliorate the state of the institutions for the reception of lunatics, that, being of a general and common-place character, may be here very well passed over, the author considers the questions, *Is insanity curable, and in what proportion? Is insanity as susceptible of cure as other maladies? Is insanity an increasing malady? Is insanity a prevalent malady?* and, *Has insanity increased?* He then treats of the *consequences of erroneous views of insanity, in reference to lunatic asylums; on the influence which the localities of lunatic institutions have on their results. Remarks on the condition of the epileptic, fatuous and idiotic. Is religion a cause, or an effect, of insanity? On the efficacy of religious instruction of lunatics; suggestions respecting legislative regulation of lunatic asylums:* and, in an Appendix, he gives some accounts of the principal lunatic asylums of Great Britain, comprising, especially, observations relating to the results of medical practice in them, and their domestic arrangements. These are treated with the view to instruction of the public, as well as the profession, respecting those matters; and therefore the author states that he has "preferred a familiar form, avoiding as much as possible all technical language and medical reasoning."

The popular prejudice that insanity is commonly incurable, is first opposed; and then a record is adduced, showing the results, in this respect, in the British, French, one Italian, and four German, institutions for the reception of lunatics. We find a great diversity in the proportion cured in the several establishments just designated; but this seems to depend, in a great measure, on other causes than a difference in the medical treatment, localities, or domestic management: as, in some, only recent cases, uncomplicated with other diseases, are admitted; in others, both old and recent cases, as well as fatuity from epilepsy and other causes. We cannot follow the author through his observations and reflections on those points; but we must notice one remark which closely interests the medical practitioner. The *Retreat* at York, (which is appropriated for the reception of members of the *Society of Friends*,) the author says, "excels every other asylum for lunatics in moral qualities; but, in the number of absolute cures, it is not on a par either with the London or Paris hospitals; and, in this respect, has much about the same relation to the cures in the former as Charenton has to those in the latter, and possibly for a similar reason, viz. that physical remedies are too lightly regarded, and therefore too little employed." The proportion of cures to the total of cases in the Retreat, up to 1811, was 36 in 100. In the Newcastle Asylum, which receives the same description of cases, and where medical means are more fully tried, the proportion is much greater.

When treating of the susceptibility of cure of insanity, the author says, he is convinced, from personal and collateral observation, that "it admits of cure in a ratio equal with almost any disorder marked by as strong indications of morbid action in the corporeal system." In support of this assertion, he observes that Willis, in his evidence before a committee of Parliament in 1789, stated "that nine out of ten cases of insanity recovered, if placed under his care within three months from the attack." Some doubts were however entertained of the truth of this statement; but it appears that the proportion of cures at the Salpetrière, a *public institution*, is but little inferior to that mentioned by Willis. Yet Dr. Burrows considers that the utmost success hitherto recorded falls short of that which is attainable; and his reason for thinking so is, that the proportion of cures, upon an aggregate of about three hundred cases in his own practice, exceeds any yet announced: that on the whole being 81 in 100; in recent cases, of 91 in 100; and in old cases, of 35 in 100. The number of deaths is 22.

"It may be necessary, by way of explanation," says Dr. Burrows, "to add, that some of these cases were treated in an establishment for lunatics under my own superintendance; but most out of it. Could one class be contra-distinguished from the other, perhaps it would be more satisfactory. But this is impracticable; because many were, at one period, at home or in lodgings, and at another in the establishment; others again were removed from it before a cure was completed. Still nearly all underwent medical treatment; and, except eight, none were lost sight of till the final event was ascertained.

"Several of the patients were in a state of fatuity, idiotcy, or epilepsy, complicated with mania; some, when I was consulted, were in the last stages of acute diseases, such as inflammation of the brain, pulmonary consumption, &c. where the mental derangement was symptomatic delirium, the precursor of death. The admixture of such hopeless cases, while it much diminishes the ratio of cures, has increased that of mortality infinitely beyond the proportion which would have attended cases of pure insanity.

"Candour impels the remark, that, whatever share of success has rewarded my humble efforts, it has probably been much favoured by an unusually large proportion of recent cases; which, as we have seen, always afford the fairest prospect of a happy event."

This renders the author's assertion respecting the curability of insanity in relation to that of other diseases less improbable than it might at first sight appear; but we have no precise grounds for a comparison, and the decision of this matter is not a subject of much importance.

Dr. Burrows acknowledges, that "the more polished, the more artificial the people, and the more prone to insanity:" yet he is not disposed to consider that it has become more prevalent of late years in England than it was at an earlier period, beyond what is explicable by circumstances distinct from our progress in the respect above alluded to; although the register of lunatics of the Commissioners for licensing mad-houses presents evidence of a rapid increase of this malady. From the years 1775 to 1779 inclusive, the whole number registered was 1783: from 1810 to 1814, it was 3647; a difference far beyond all proportion to the increase of the population. We shall add the number in the intermediate decades: from 1780 to 1784, 1893; from 1785 to 1789, 1892; from 1790 to 1794, 2292; from 1795 to 1799, 2242; from 1800 to 1804, 2463; from 1805 to 1809, 2271. The progress of these numbers is not regular; and in two instances there is a little retrogression. It would, perhaps, not be difficult to find satisfactory reasons for these circumstances in our political and social state. The increase was but very little during the first three decades, which comprise a period of remarkable national prosperity; and one of the retrogressions happened in that corresponding to the state of peace with France: we may add, too, that the first great increase took place during the turbulent period from 1790 to 1794. Dr. Burrows attributes this to the late King's first illness; "an event," he says, "which induced a universal and deep sympathy, and produced also an unusual interest in the condition of all similarly affected; and this effect was much heightened by the publishing of the examination of the attending physicians, on the nature and probable issue of his Majesty's malady. The result superadded an unprecedented number of entries to the fourth lustrum, which commenced in 1790."

A court-physician to Louis the Fourteenth would have felicitated himself on so happy an imagination. The defective harvest in 1800 is adduced to explain that in the sixth decade; and of this we cannot dispute the propriety: Ireland and France furnish evidence in support of it. In 1815, the number admitted into the Cork Asylum rose from 74 to 120. Bread was at an exceeding high price in France in 1816, and the number admitted into La Salpetrière became, the ensuing year, double what it had been in any former year since the Revolution. In allusion to a former remark, we should state, that Dr. Burrows thinks the mere occurrence of any thing which elicits the public attention to the state of lunatics, gives rise to the return of many in the registry that would otherwise have escaped it; and hence, he thinks, Mr. Wynne's Act, which passed the Parliament in 1808, will account for the increase in the last decade. We do not exactly agree with the author in this instance; being disposed to attribute the increase alluded to principally to the sudden fall of national prosperity, which obliged every individual to make extraordinary exertions to preserve the rank in society he had previously held or expected to attain, and plunged multitudes into abject poverty, misery, and want. The effects of this are subsiding: new levels in society have been formed; and the number of lunatics has for two or three years retrograded, as appears by the register of the Commissioners, and that of the pauper-lunatics in Mary-la-bonne parish, which has been nearly the same during the last five years, although it is probable that the pauper population has increased. We must not certainly calculate on an increase of it; because the great addition to the population of this parish consists principally, if not wholly, in persons above the rank of paupers. Unhappy Ireland does not partake of these results: there insanity goes on increasing with the misery and desolation of the people.. But we have no proper grounds for the discussion of the question, whether insanity has increased in consequence of the progress of civilization; for the records which Dr. Burrows adduces with this view do not extend back half a century. There are, too, a multitude of circumstances which render it difficult, if not impossible, to draw any correct inferences from the records we have presented to us, resulting from want of accuracy in the construction of them.

The question, *is insanity a prevalent malady?* is one that does not admit of a precise reply. We do not see the utility of the discussion of it. All that we can say is, that this malady bears such a proportion to all other diseases, or to any particular disease; and what one person may choose to call prevalent, another person may think rare. The register of the Commissioners makes it appear that, in 1800, the proportion of lunatics was 1 in 7300, in the population generally. But this calculation appears not to have been accurate; and Dr. Burrows makes it appear, that the proportion, in 1810, was 1 in 2000. The question, *has insanity decreased?* might have been involved in a former one. Our limits will not permit us to dwell further on these points: those of our readers who feel interested in them, are referred to the work itself.

On treating of the consequences of the prevalent erroneous views in reference to lunatic asylums, the author first shows, that the notion that insanity was rapidly increasing has led to evil, in the first instance, in the construction of the county lunatic asylums, most of which are much larger than is requisite, and, consequently, cause an unnecessary expense in the support of them. Bethlem and St. Luke's are, indeed, not filled at the present time; yet it must be acknowledged that this is better than a want of space for the reception of patients: and the author remarks, "although the projectors of particular asylums may have committed the fault of building them too large, yet their beneficent views may be equally gratified by converting this very error into a means of extending the benefits which such institutions are intended to confer. Three essential purposes, to which an unoccupied portion of an asylum may be applied, are prominent: 1. To a better classification of the cases than is usually practised; 2. To the reception and permanent lodging of a certain number of incurables; 3. To the reception of cases of epilepsy, idiotcy, and other forms of mental derangement, which are too generally excluded." The expediency of each county having its own asylum, is of doubtful propriety. It renders them necessarily small, and thus prevents the possibility of resorting to measures which are calculated to be of much benefit. If several adjoining counties were to unite their funds, institutions might be formed like that at Bayreuth in Germany; where so large a tract of ground is enclosed, that all the lunatics who are not furious have sufficient land given them to cultivate to occupy them the whole year; and, such is the influence of habit and example on them, that it is never found necessary to resort to violence to make them pursue their daily labour. Such means as this must be powerfully conducive to the restoration of health, especially in those who have become convalescent.

We pass over the next section, which consists of remarks on the local situation and construction of asylums, as not adapted for analysis in this place. The following one, too, consists chiefly in propositions for the more proper accommodation of insane epileptic, fatuous, and idiotic, patients in lunatic asylums; the intermixture of these with other species of mental derangement being injurious to both classes in various ways.

*Is religion a cause, or an effect, of insanity?* is the question next discussed. The reader need not be alarmed; the author does not intend to hint that *religion* may be an effect of insanity: the curious allusion this question presents arises from an abuse of the word; he means *false and fanatic theology*. This is too delicate a point to be discussed in the space we have yet remaining in the limits of this article. The author thinks it never arises from Christianity, until doubts are admitted about the truths of some of the dogmas that constitute it; and hence it is commonly found, when referable to this source, to have occurred in persons who have gone over from one sect to another, and have confidence in neither. We have occasion to remark here, that the author's inferences go beyond his arguments. He says,

"It has been with some a favourite hypothesis, that insanity *frequently* originated in the theological tenets peculiar to certain sects; and that persons professing a form of devotion free from controversial intricacies, and therefore such as might be comprehended by the plainest understanding, as, for instance, that of the Quakers, would be *entirely* exonerated from this severe affliction."

Such an inference would only be admissible on the argument, that insanity *always* originated from religion.

Several interesting remarks are adduced in relation to the subject of this section; and six cases are related to illustrate this point, in five of which the patients were females; and it seems that it is chiefly persons of this sex who become mad from fanaticism, or are fanatical in their madness.

The author's suggestions respecting legislative regulation of lunatic asylums, and the contents of the Appendix, will be found interesting to those who devote their attention to this part of medical police, but are not adapted for analysis. We need hardly say, that the work, altogether, comprises much interesting information on this subject, for our extracts must have rendered this evident; and much of this information is such as could not have been procured without considerable exertions. We doubt, however, whether it is qualified to produce any remarkable benefit. The evils pointed out in it cannot be unknown to the legislature, and continue to exist only because it is more difficult to remove than to discern them. The propositions of the author for the improvement of this point of medical police do not appear to us to comprise any original suggestions of much importance. The question may indeed be proposed, with apparent propriety, whether it is not better, at least as far as the medical part of the administration is concerned, to let matters proceed in their present train. Now that the foolish metaphysical notions about insanity prevalent in the last century are becoming obsolete, the treatment of this disease cannot fail to keep pace with the improvement of our pathology of it; and this must result from precise observations and particular inferences, not from any suggestions of a general and indeterminate character.

As this work is likely to be perused by the public, we are sorry to find it present a very inferior specimen of medical literature, as far as literature is concerned. It would not be difficult to select from our medical writings of the last half-century several works which might serve as models for the literary composition of treatises on physical science; and the simplicity and elegance they possess have become characteristic of the generality of modern English medical works; but the style of that of Dr. Burrows is awkward and embarrassed: the chief fault in it, however, is the frequent introduction of new words, either original with him or adopted from affected writers of an inferior character, that were not wanted in our language for the expression of any idea, and that are often much less euphonous than those they are made to supersede: as, *succumb* for *submit; punition* for *punishment; to individuate* for *to particularize; brainular* for *cerebral; debet* for *residue; defunction* for *death; decrement* and *increment* for *decrease* and *increase; ratiocinating* for *reasoning; unfortunates* for *unfortunate persons.* We also find several expressions devoid of precise meaning, as *hyper-perfection, enduring man, impracticable malady.* Though we cannot agree with Madame DE STAEL, that these vices of composition indicate a barrenness of ideas, we regret to find them in this work; for they must subject our literature to the severe censure passed on it by JOHNSON in the judgment of many respectable members of the community, who are not acquainted with the specimens of elegant composition that might be selected in abundance from our modern writers. There was, a few years since, some danger of our having a flowery style come into use; but one writer alone has wholly obviated it, by the disgust which his poetical rantings has excited in the minds of all men of good taste.

---

***Letters to the President of the Associated Apothecaries and Surgeon-Apothecaries of England and Wales, on the present State of the Practice of Physic and Surgery. First Series: intended to give a Comparative View of particular Systems of Medical Education; to consider the Separation of Medicine from Surgery; to estimate the Claims of the General Practitioner; and to propose a more respectable Mode of remunerating his Attendance.*** London: Burgess and Hill. 8vo. pp. 77. 1820.

[The following critique is the production of a gentleman who exercises the duties of a surgeon and apothecary in the suburbs of London. As it is the interests of this class of the profession to which the "Letters" expressly relate, we are confident that those of our readers who range in it will be pleased that we have thus availed ourselves of the talents of one of the most enlightened of their peers in the consideration of the merits of their cause; and we think the gratification we have ourselves experienced from the perusal of his remarks, will be participated by the profession in general. The insertion of them in this Journal would alone show that we approve them; but we regard them with feelings of more than simple approbation, and think this production the best argument that has been advanced to show the propriety of the claims which it is the object of the "Letters" to advocate.—EDIT.]

WE are glad to find that this important subject is on the eve of being again considered by the profession and by the public. The improvement of medical science, and consequently the welfare of the public, are both so closely connected with the respectability of those who practise medicine, that we are sure, not only medical men, but the public in general, will join us in thanking the author of these Letters, for having, with the honourable feelings of a gentleman, and the dexterity of one not unused to wield the pen, made manifest some of those inconsistencies which must of necessity exist, in the progress towards perfection of a mixed system, which, like the British constitution, has been the child of expedients, and altered in its institutions, according as the wants of an improved state of society have shown the inefficiency of old laws, and made new ones necessary.

The present publication is, it seems, only the first of a series of communications, which are intended to show the faults of existing medical institutions, and to point out wherein the system requires to be improved. But we should have been glad if the author had given us some insight into his general plan, instead of plunging at once into the middle of his subject; because he would have thus avoided giving some parts of his argument the best chance of being erroneous, by setting out with a clear conception of the object which he wished to gain.

The present series of Letters begins with an account of the advantages which have followed the passing of the Apothecaries' Act in 1815; and an opinion, that it will materially benefit the middle and lower classes of society, by advancing the surgeon-apothecary and apothecary in the scale of improvement and usefulness. The mode in which the general practitioner is remunerated for his services comes then into discussion, with an intention of showing, that, whilst it is defective and unjust, it degrades the character of him on whose respectability much of the health and safety of a large majority of the English people depends. The author then recommends that the general practitioner shall be remunerated by a fee proportioned to the finances of the patient.

A letter follows on the present system of medical education; and a comparison is drawn between the collegiate education of the higher physician, and the apprenticeship which forms the prominent part of the plan on which the general practitioner is educated.

A fourth letter, on the impropriety of dividing medical practitioners into physicians and surgeons, closes the series.

It will be easy to show, that the present division of medical practitioners into physicians, surgeons, general practitioners, druggists, &c. did not exist formerly, but has gradually arisen to meet the exigencies of the times and the wants of an increasing population. Anciently there was but one kind of medical man, the physician. (See Hippocrates' letter to his herbalist to send him simples, with directions how to preserve them, &c. as also, in the book Περι ευχημα, a description of a physician's shop.) Galen, too, travelled to collect medicines. It is stated, in Dr. Hodge's *Vindicium Medicinæ*, &c. which was written much earlier than the year 1678, that physicians formerly made their own medicines; and at that time the most dreadful contentions existed between the Galenists and the pseudo-chymists, as Dr. Hodges calls them: in fact, even at that early period, the apothecary, who was only a druggist, which the physician had tolerated to save himself trouble, had made himself so useful to the physician's patients, as to excite his jealousy in a very high degree; but it was not until about the year 1678 that a determined civil war raged between the physicians and the apothecaries, and gave birth to many curious publications, which have now become exceedingly interesting, from the picture which they draw of the state of physic at that period.

The apothecary had become by this time an important member of the medical profession, and the physician vainly lamented that he had consulted his own ease by tolerating his existence at the expense of his own emolument; not seeing, as he might have done, that the first rise of the pharmacopolist, and his conversion into a practising apothecary, was owing to the increasing wants of the people, and the demand for the greater supply of medical men than the ranks of the physician could afford.

A history of these contentions would be exceedingly amusing. The anger and ignorance displayed on both sides, the mutual revilings, the prison-house secrets which thus got vent, and the vain attempts of the physicians to interfere with the natural order of commercial operations, excite in the reader a hearty laugh at every stage of the dispute. We cannot, however, refrain from quoting a few words from some observations which we extracted a few years ago from the writings of those disputants. The most grievous charges were brought by the physicians against the apothecaries. It is said, how they trifled with the lives of the people, by their ignorance, and from their thirst of gain; how they used medicines contrary to the prescriptions, myrtle-leaves for senna, sheep's lungs for foxes' lungs, the bone of an ox's heart for that of a stag's heart, &c. and otherwise falsified the grand compositions of the London Dispensatory, by using decayed drugs, making diascordium of honey and bole-armeniac, and by directing their shopmen, when he complained of having no good rhubarb, to put in as much again as was ordered of what he had: how they multiplied their bills and medicines beyond reason, and decried physicians who would not order for their advantage before that of the patient; with the intention of calling in those "careful and painful doctors who will pay two or more visits in a day, and write a new bill every time, till they have planted the cupboards and windows of the patient's house with glasses and gallipots;" how they charged high, and put "a pint of julep, which is to be taken at four times, into four parts, and carrying but one at a time, made it come to ten, twelve, or more shillings;" and took care to make extra charges at the foot of their bills for bezoar, pearls, gold, and rich cordials; how they made presents to physicians to help them to customers; and how they aimed at the ruin of the physician, by opposing and traducing him, &c. and, which was the unkindest rub of all, by calling the sick their patients. (See Dr. MERRETT's *View of the Frauds of the Apothecaries*, published in 1669.)

It would seem, however, as if the house of the physician was unhappily divided against itself; for there were some who, either conscientiously or for the sake of their own interests, encouraged these encroaching and abandoned apothecaries; for, in the *Medela Medicorum*, published in 1678, it runs thus: "For there are not very many patients to whom an apothecary, and that for reasons hereafter to be told, is not first called, who, being consulted what to do, if he thinks he can carry on the business privately, he undertakes it, goes smoothly on, makes no noise at all, and takes the whole advantage to himself; that prey being caught in his net, he makes sure of it. But, if difficulty occur, and by his applications and advises bad symptoms are not taken off, but increased, the patient growing worse and worse, then either some friend of the patient's sends, through good will, an honest physician to take care of him; whom the apothecary not finding fit for his purpose, tells the patient, or his near relation or friend, privately, and that with a good deal of seriousness, that, although that physician they employ be an ingenious man, (therefore he is loath to speak any thing against him,) yet truly, for the good will he oweth to the patient, his worthy friend, he cannot but acquaint them that the doctor mistaketh the patient's case; this or the other medicine he prescribes, is not proper for him: thus ceaseth not, till he, by his cunning jugglings, gets him shuffled off; all which he pretends to do privately, as their intimate friend, and therefore begs their concealment: which being done, he gets a fair opportunity of sending for whom he pleaseth; and, having several in his eye, some fit for one purpose, others for another, so that, if he thinks the patient will linger for a while, then he gets them to send for one (whom he highly commends), whom he is sure is fit for his purpose; viz. will make large prescriptions, order rich cordials, &c. But, if he think the patient is hasting away, and that he would have the thorn taken out of his heel, then he intimateth another to them, whom they must by all means send for. This is his *hackney doctor*, (of which the patient and his friends are ignorant,) who is in league with him on purpose for such jobs; and this doctor forsooth, for the advantage of a fee or two, (many he cannot have, because the patient hastes away,) and, in hopes of more work upon the like occasions, approves and commends all the apothecary has done; tells them, he has acted as much as could well have been done; if he or any other physician had been called before, they could have done no more: so patronizeth what the apothecary has (perhaps ignorantly enough) done. Thus the patient is artificially killed by the apothecary, and the whole as cunningly buried by his journeyman doctor. By such juggling tricks, many patients' lives (God knows) are cast away; while these two carry on a colleaguing, deluding design, a wickedness too gross to be fathered (one would think) upon any of the pretenders to this in itself noble faculty, and too palpable to be connived at.

"And, as some apothecaries have their hackney doctors, who, being of a meaner employ, are ready at any time to do the apothecaries' jobs, so also some doctors, who have more work, have their *covenant apothecaries*: such, I mean, as, to the great disgrace of the faculty, by agreement, go snips with the apothecary, perhaps five shillings or ten shillings per pound. How high such apothecaries' bills must be, the patient is made to know to his great cost: in order to which, they have the knack of procrastinating cures, by exhibiting some palpable and specious, but ineffectual, cordials, chips in broth, &c. enough to amuse, but too little to effect any thing considerable, otherwise than to swell the apothecary's bill. And, the better to bring their patients to these their covenant apothecaries, and, once got, to keep them thereto, they mark some ordinary medicine (some small inconsiderable thing, if the trick was known), with a different character, or else have some addition to a trite medicine, which, forsooth, must then be a nostrum, and to be had nowhere else but at the aforesaid apothecary's shop."*

But we must leave these *nugæ canoræ*. It is sufficient to say, that the wants of the people required the apothecary, and therefore he maintained his ground, and increased in reputation, in spite of all opposition.

It was not however until the latter end of the last century that the medical duties of the apothecaries became so extensive, as to require for the profession a further division of labour; but, about that time, a new order of medical men, the druggists, began to multiply, and the extension of their numbers has been indeed most rapid. The majority of general practitioners, we believe, consider their progress to have been appalling as well

---

* Page 23 *et infra*.

as rapid; because they think that an improper encroachment has thus been made upon their just rights. Whether this be true or not, remains to be seen; but hence arose a new feud in the profession, which may be thus described: By the progress of opinion, the apothecary, who was formerly only a druggist, had become a physician; and, as the old apothecary was still more than ever required, the druggist took possession of his vacant stool, and thus excited the same jealousy in the new physician as the encroachments of the old apothecary had done in the mind of the old physician. The new physician has however, in one respect, shown more wisdom than the old one. He has regretted, it is true, the loss of emolument which this change has occasioned, but he has repined in secret, and has not held himself up to ridicule by a vain public opposition to what was unquestionably produced by the uncontrollable march of that cause from which springs all commercial regulations. The apothecaries were certainly wrong for becoming grand, and shutting-up their open shops, because they hastened the sad catastrophe; but we believe that nothing would have prevented its occurrence.

Such we consider to be the mode in which the present classes of medical men have arisen. It is a patch-work system, and therefore may reasonably be supposed to be defective; and this leads us to the main object of the author in writing the present series of Letters, namely, the showing how improper the present mode of remunerating the apothecary is. We fully agree with the author in saying, that the charging an exorbitant price for medicines, and thus being paid for professional skill by an as it were fiction in law, is hurtful and degrading: indeed, we know that it is a constant source of chagrin to every gentleman who is engaged in the profession of a general practitioner. It loudly calls for alteration; and we hope that the commencing discussion will not cease, until the payment of the general practitioner, by means of a proportionate fee, instead of by a degrading overcharge of medicines, which may or may not be necessary, has become general. In the expectation that such may be the result, we have thought it right thus to clear the ground a little for the approaching combatants, by putting into their true light some important preliminaries to the discussion.

We are sorry that we are prevented from noticing the separate arguments of our author as they deserve: as they are well put, however, and as they will therefore be read by all the profession, we are the less anxious to remedy our omission. Not that we quite agree in all his conclusions; and therefore we shall make a few observations, in the way of friendly discussion, upon two points, namely, the value of apprenticeships, and the division of professional practice into medicine, surgery, midwifery, &c.

With regard to apprenticeships, the author presumes, and we agree with him, that the collegiate discipline of the physician, and the apprenticeship of the general practitioner, form the leading points of difference between the education of these two members of the profession. But, are they both equal in medical knowledge, as the author infers? We apprehend not; and we say this with all due respect for the apothecary, to whose ranks we belong. We consider the reason of this difference to be, that the collegiate education of the one is good; the apprenticeship of the other is bad. No man can study medicine with advantage, unless his mind have been previously stored with general knowledge, and he has learned the difficult art of comparing this knowledge, or what is called the *art of reasoning*. Does a residence at college give this? We think it does in an eminent degree. Does an apprenticeship? We think not at all, as they are at present conducted. In the first case, a boy is kept at general study until the age of twenty or more, and the fine period between puberty and manhood is thus made the most of; so that the student begins his medical education under every advantage. The same thing cannot be said for the future general practitioner. His general education stops just at the time when the mind is beginning to expand; and he is doomed to waste five most important years, either in idleness, as generally happens, or in the gaining of mere empirical information, which must, for the most part, be afterwards unlearned.

But, if apprenticeships were ever so well constituted, we should still say, that more general study, and, if possible, collegiate study, should precede them; and that a considerable interval of attentive application to anatomy, and the other preparatory medical studies, should pass, before the student can reap due advantage even from the limited routine of an apothecary's shop. As it is, we know from experience, that an apprentice comes ignorant to his post, and in general does nothing but gain something of a doctorial air while he continues there.

In short, we consider that both ranks should be educated in the same way; that the apothecary should, if possible, go to college, and the physician be indispensably required to enter the apothecary's shop during the middle or latter part of his pupillage.* In this way, if further improvement were made

* We say indispensably, because it is quite evident that physicians are generally very ignorant of the pharmaceutical branch of the profession.

in the more direct medical education, a new race of practitioners would spring up, far exceeding in learning and usefulness the best physicians of the present day.

We think, too, that the author is in some degree wrong in his reasonings on the arbitrary exclusive division of practitioners into physicians, surgeons, &c.; and his error, as it appears to us, consists in drawing an unauthorized conclusion from the fact, in which every sensible man agrees, that the different branches of medicine must be studied at the same time; and that no surgeon can be competent to practise unless he is a good physician. Anatomy and physiology are the groundwork of all medical and surgical science; a general, and even extensive, knowledge of pathology is also requisite; but, when once this has been obtained, the public will be, as it has been, materially benefitted by one man directing his attention almost exclusively to surgery, and another to midwifery, &c.: nay, even so difficult is the attainment of a perfect knowledge of even one set of diseases, that much good results from individuals taking much smaller divisions of the healing art for their almost exclusive study. In some cases, as in oculists, dentists, &c. this subdivision is designedly made: in others, as in the accidental direction of a physician's attention to fevers or chest-complaints, or the like, he acquires a much more accurate knowledge of such diseases than any physician, whose opportunities have not been so extensive, can possibly acquire.

We hope the author understands us; and then we are sure he will take our strictures in good part. His Letters are likely to do much good; and we hope soon to receive a second series from his pen. We would however, in parting, recommend him to give book-references to the many excellent quotations with which his pages abound.

---

***Observations on the Properties of the Air-Pump Vapour-Bath, in the Cure of Gout, Rheumatism, Palsy, &c.*** By M. La Beaume, F.L.S. &c. 12mo. pp. 275. Highley and Son; 1819.

***Remarks on the History and Philosophy, but particularly on the Medical Efficacy, of Electricity and Galvanism, in the Cure of Nervous and Chronic Disorders, &c.*** By the same. 12mo. pp. 373. 1820.

It is a very interesting circumstance in the history of the *Materia Medica*, that the date of the regular use of many of its most active agents relates to a period several years subsequent to that of the original discovery of their efficacy; and that, during the interval here indicated, they have been either almost totally disregarded, or only employed by some of the more zealous or adventurous practitioners of the medical art. This remarkable occurrence presents, when referred to its origin, a lesson on which we cannot too deeply meditate: it teaches us that the enthusiasm which has led men through all the toils of original research to new discoveries, and without which these would not have been attained, has often been the most certain means of preventing their merit being generally acknowledged. The intemperate zeal with which they were originally proposed as remedies, necessarily led to disappointment and disgust, from the results of injudicious application of them; and, for a time, wholly prevented their value being properly investigated, by causing them to be regarded with injurious prejudice by the persons best qualified to form a correct estimate of their merit: and of this we have an example, in the conduct of the generality of the profession in respect to one of the measures advocated in the works before us.

The use of the air-pump vapour-bath was proposed several years since by a physician of considerable talents, who at the same time adduced satisfactory evidence of its powerful efficacy; but he seems to have evinced more zeal in its favour than his contemporaries were disposed to participate with him; and this remedy, which reasoning *a priori* would lead us to expect great effects from, had become almost totally neglected in England: not from its having failed to produce what it was stated to be qualified to do, but absolutely from the want of a proper trial of its powers. It has, however, been adopted by other nations: the French commonly resort to it; and Professor HUFELAND, of Berlin, has lately endeavoured (in the *Journal der Practischen Heilkunde*, Mai 1819,) to elicit the attention of the faculty more generally to this remedy, by his warm expressions in favour of its use in several of the affections for which Mr. La Beaume has also employed it with the most gratifying, and often extraordinary, results.

The great efficacy of the vapour-bath, either partial or general, in many diseases, has been universally acknowledged: now, this apparatus combines with the measure just indicated the power of producing a rarefied atmosphere round the whole or part of any of the limbs, to an extent that may be easily regulated according to our desire, and which may also be maintained for an indefinite period. The machine possesses, too, the conveniences of simplicity and portability in its construction. It consists of a metallic cylinder, having an orifice at one extremity adapted for admitting the limb, which may be closed by means of a piece of oiled silk folded round

2

it by a bandage; at the other end of the cylinder is placed a stop-cock, which receives a tube coming from the top of a vessel containing water, which is heated by spirit-lamps (one or more of which may be employed according to the degree of heat required), and transmitted through the stop-cock into the cylinder. A thermometer is attached to this part of the machine, to show the temperature of the vapour it contains; and this fluid is permitted to pass out of the vessel by an escape-valve and flexible tube. When the limb has been sufficiently exposed to the warm vapour, an air-pump attached to the cylinder is put into action, and such a state of exhaustion of it produced as the feelings of the patient, or the nature of the disease, may render advisable. The whole process usually occupies about an hour.

We have not ourselves had opportunities for the immediate observation of the effects of this remedy, but we cannot doubt that it is one of no ordinary value, and hope that it will ere long receive the attention it appears to merit. The chief objection to the use of it, amongst the two higher ranks of the profession especially, is the necessity that some person well acquainted with the principles of its action, and possessing some knowledge of physiology, should superintend the application of it. Physicians cannot do this; and surgeons have not the inclination, even had they sufficient leisure. This objection is now obviated as far as the practitioners of London are concerned, by Mr. La Beaume having adopted the employment of this remedy as part of his duties; and we feel much pleasure in being able to add our testimony to that of many of the most eminent medical practitioners, in favour of the excellence of his qualifications for the profession he has assumed. It is, however, in the application of electricity and galvanism to medical purposes, that these become especially beneficial. Nothing has tended more to excite the disgust of the medical practitioner for these remedies, than the assumption of the application of them by empirics; and it is from the same cause, and the neglect of due attention to them by the members of the faculty, that the value of them has been rated in, apparently, far too low a manner, and that the unfounded fears of their agency, prevalent to a great extent, have derived their origin. Mr. La Beaume is well acquainted with what is known of electricity and galvanism, and is well informed respecting the physiology of the human body; and he evinces much discrimination and judgment in his mode of employing them. He has three ways of producing the galvanic influence: one, for affecting the system more generally, in which he makes the epigastrium and the upper part of the spine form the points of communication, the effects of which are highly invigorating in many cases of disease; the second, in which the former method is combined with an especial local influence, as when the ear or any other part is made the medium of forming a chain with the epigastrium, one of the hands, &c.; the third is more expressly local, in which the points of communication are made on different parts of a limb, near the eyes, in the auditory canals, &c. When applied in the two latter ways, only instantaneous shocks, in rapid succession, not so violent as to cause pain, but only a sensation of heat or throbbing in the points of communication, should be produced: a continuance of the galvanic influence for a few seconds would here produce head-ach, vertigo, &c. He has obtained results from these measures that show them to be worthy of being more generally employed than practitioners in general are disposed to believe. The general indications for their use must be evident, and our limits will not permit us to enter into particulars respecting them; yet we must state that the cases here related show them to be efficacious in many affections to which they have not been generally considered applicable with so much benefit: this is especially the case in regard to torpor of the liver and dyspepsia from debility, more particularly in persons whose nervous system has been rendered comparatively inert by long-continued sufferings or too great mental exertions.

We shall only add to the foregoing remarks, that we think the public is much indebted to Mr. La Beaume for his meritorious exertions to place the use of those remedies in their proper sphere; and that it should be a cause of much peculiar gratification to medical practitioners to know that they may resort to his aid, in cases where they consider those remedies applicable, with the assurance that their advice will be conformed to in a judicious manner, and with confidence that they so far consign their patients to the care of a gentleman of liberal education, whose conduct has done honour to his profession.

